# Supplementary material for: A MEC-2/stomatin condensate liquid-to-solid phase transition controls neuronal mechanotransduction during touch sensing
Source: Nat Cell Biol. 2023 Oct 19;25(11):1590–9. doi: 10.1038/s41556-023-01247-0 (PMC10635833; doi:10.1038/s41556-023-01247-0)
Supplement: Supplementary file 1 — Supplementary Notes 1 and 2; and legends for Supplementary Videos and Tables. [file 41556_2023_1247_MOESM1_ESM.pdf]

nature cell biology

Article https://doi.org/10.1038/s41556-023-01247-0

A MEC-2/stomatin condensate liquid-to-solid phase transition controls neuronal mechanotransduction during touch sensing

In the format provided by the authors and unedited

Contents

1 Supplementary Note 1: Optical Trapping **2**

1.1 Single trap stress relaxation of protein condensates *in vitro* . . . 2

1.2 Dual trap active microrheology of protein condensates *in vitro* . . . 3

2 Supplementary Note 2: Plasmid design and genome engineering **7**

2.1 Expression of wild type and R385H *mec-2* and *unc-89* for *in vitro* purification . . . 7

2.2 Expression of wild type and R385H *mec-2*::mCherry *in vivo* . . . 8

2.3 Generation of the *u37* allele in *mec-2* . . . 8

2.4 Expression of *mec-2* in hypodermis . . . 9

2.5 Generation of the FRET constructs . . . 9

2.6 Promotor trapping of *unc-89* . . . 10

2.7 Tagging of *unc-89* SH3 domain . . . 10

2.8 Insertion of TEV site in *mec-2* . . . 10

2.9 Mutation of PRM in *mec-2* . . . 10

2.10 Generation of (mEGFP(loxP)::AID knock-in)*unc-89* . . . 11

2.11 Generation of split wrmScarlet *unc-89* . . . 11

2.12 Generation of the conditional and constitutive *unc-89* knock-out . . . 12

2.13 CRE/loxP and AID degradation . . . 12

3 Supplementary Videos **13**

4 Supplementary Data Tables **15**

Supplementary Material

1 Supplementary Note 1: Optical Trapping

1.1 Single trap stress relaxation of protein condensates *in vitro*

Freshly prepared MEC-2 condensates (t=0 h) showed a rapid and almost complete relaxation, indicating that naive, liquid-like MEC-2 condensates cannot store mechanical stress. This fluid-like signature remained largely unchanged for the first 24 h after condensate formation (Extended Data Fig. 8c). Only after 48 h, MEC-2 showed a significantly slower relaxation time scale despite retaining a constant stiffness (Extended Data Fig. 8d), reminiscent of a glass transition with an age-dependent increase in viscosity (Extended Data Fig. 8e,[1]). Interestingly, we found the same viscoelastic behavior with the condensates composed of mutant MEC-2 (R385H), suggesting that MEC-2 maturation *in vitro* is a slow process that does not depend on the presence of arginine at position 385 (Extended Data Fig. 8f-h).

Condensates in the single trap configuration were prepared as described in the methods and settled onto the coverglass and strongly attached to it, but were prevented from wetting and thus remained in a droplet shape (Extended Data Fig. 8a). Stress relaxation tests upon step-strain indentations onto the droplets were carried out by addressing a square oscillation protocol to the trap. The bead pushing force against the droplet arose from a change in light momentum that was detected with our direct force sensor. Upon the pushing event, the force peaked to F_p and relaxed exponentially to a resting force, F_0, which arose from surface tension (Extended Data Fig. 8b). Upon pulling, we observed a nearly symmetric peak followed by a relaxation curve, with opposite sign, due the bead being adhered to the droplet. The force-time curves were fitted with an exponential function that describes the time-dependent decay in force as follows:

$$F(t) = F_0 + (F_p - F_0)e^{-t/\tau} \quad (1)$$

Parameter τ corresponds to the relaxation time constant of the Maxwell element and equals $\tau = \eta/k$, where η and k are the viscosity and the stiffness of a dashpot and a spring in series[2]. We observed an increase in τ for older condensates (throughout a time frame of 48 h), indicative for Maxwell fluids undergoing hardening upon maturation (aging). This is consistent with a higher ability to store mechanical

stress for a longer time (see Main Text, Extended Data Fig. 8c,f), for time scales that are smaller than τ. In turn, the spring stiffness was measured as $k = (F_p - F_0)/d$, where $d = 100$ nm is the amplitude of the square oscillation. We did not observe an increase in k in between the different conditions tested. Thus, droplet ageing did not seemingly affect their stiffness, as measured in this experiment. We noticed that this lack of stiffening is similar to the ageing properties reported by ref. [1] which reported invariant elasticities during the maturation of five different condensates. We note though that our measurement represents the structural stiffness and not necessarily the elastic plateau modulus derived from an active microrheology routine (see below). Importantly, we did not observe a statistically significant change in this assay between condensates composed of pure wild type and mutant MEC-2. Thus, MEC-2 forms rapidly relaxing condensates that undergo a relatively slow ageing process.

1.2 Dual trap active microrheology of protein condensates *in vitro*

To further characterize the frequency dependent response of MEC-2 condensates and their derivates, we established a dual-trap active microrheology routine. To prevent condensate adhesion to the substrate which would modify force propagation between the two traps altering the force balance in an unpredicted way, we used the surface passivation reported in ref. [3]. Only spherical condensates were found laying on the coverglass and could be detached and manipulated with the optical trap we used for the experiments. A total trapping power of $P_T = 40$ mW at the sample plane was split into two time-shared optical traps with an average power of $P_{trap} = 20$ mW each. Once two beads were optically trapped, each trapping stiffness, k_1 and k_2, was obtained by rapidly-scanning the trap across the bead using the Particle Scan routine of the manufacturer's software, as described elsewhere[4]. Importantly, the time-sharing frequency to generate the two traps at different locations was $f_{TS} = 25$ kHz, far beyond the relaxation time of our measurements, thus we assumed that the two traps are continuous during the force measurements.

We attached one bead on each opposite side of a protein droplet along the *x* direction (Fig. 5a-ii) and stretched the droplet. Trap 1, on the left hand side, applied a square or sinusoidal oscillation insults, depending on whether a stress relaxation test or a rheological measurement on the frequency domain

was getting acquired, while trap 2 was kept motionless on the right hand side (Extended Data Fig. 10a, Supplementary Video 6). Similar to the single-bead stress relaxation tests (Extended Data Fig. 8b), for square oscillations, force on trap 1 peaked at $F_p^{(1)}$ upon every pushing event and relaxed exponentially approaching a resting force, $F_0^{(1)}$ (Extended Data Fig. 10a-ii). Upon pulling, we observed a similar peak followed by a relaxation curve, with opposite sign, due the bead being adhered to the droplet. An opposite, reaction force on bead 2 was measured with a lower peak force, $F_p^{(2)}$, and was as well followed by a decay curve. We determined the steady-state resting force values after complete relaxation, $F_0^{(1,2)}$, through fitting the F-t curves to Eq. 1 and determined the resting droplet spring constant, χ_0, and thereby obtained a measurement of the surface tension, γ, according to Eq. 3 [5]. At the same time, beads 1 and 2 were displaced from the respective trap centers by F_1/k_1 and F_2/k_2. Being $d = 100$ nm the oscillation amplitude of the square trajectory of trap 1, droplet deformation, Δx_0, and thereby χ_0, read as:

$$\Delta x_0 = d + \frac{F_0^{(1)}}{k_1} - \frac{F_0^{(2)}}{k_2} \tag{2a}$$

$$\chi_0 = \frac{F_0^{(1)} - F_0^{(2)}}{2\Delta x_0} \tag{2b}$$

Using the expression reported in [5], we obtained the surface tension as follows:

$$\gamma \approx \frac{\chi_0}{\pi}(-\ln \theta_0 + 0.68) \tag{3}$$

The complex spring constant of the protein condensate, $\chi^*(\omega)$, was measured by applying sinusoidal oscillations, with an amplitude of $d = 100$ nm, at increasing frequencies $f = 0.5, 1, 2, ..., 64$ Hz (Extended Data Fig. 10a-i). Traps 1 and 2 measured the force acting onto each optically-trapped bead upon the oscillation (Fig. 5a-iii and Extended Data Fig. 10a-i). We calculated the Fourier transforms of the force signals at the driving frequency, $\tilde{F}^{(1,2)}(\omega) = \mathcal{FT}[F^{(1,2)}(t)]$, using the fast Fourier transform (FFT) algorithm in Matlab. We finally calculated $\chi^*(\omega)$ from the frequency-dependent complex spring constant of the coupled system (*trap 1 - droplet - trap 2*), χ^*_{sys}, as follows:

$$\chi^*_{sys}(\omega) = \frac{\tilde{F}_1(\omega) - \tilde{F}_2(\omega)}{2\tilde{x}_{sys}(\omega)} \tag{4a}$$

$$\chi^*(\omega) = \frac{\chi^*_{sys}(\omega)[4k_1k_2 + i\xi\omega(k_1 + k_2)]}{2k_1(2k_2 + i\xi\omega) - 4\chi^*_{sys}(\omega)(k_1 + k_2 + i\xi\omega)} \tag{4b}$$

In Eq. 4a, $\tilde{x}_{sys}(\omega)$ is the Fourier transform at frequency ω of the length of the coupled system, taken from the optical trap coordinates as $x_{sys}(t) = x_{trap}^{(2)}(t) - x_{trap}^{(1)}(t)$. In Eq. 4b, $\xi = 3\pi\eta D$ is the friction coefficient of the droplet in a viscous medium with $\eta = 1$ mPa·s. In turn, D is the droplet diameter and was measured as $D = x_{trap}^{(2)} - x_{trap}^{(1)} - \phi$, where $\phi = 1$ µm was the diameter of the microbeads. The complex spring constant captured the main features of a liquid phase separated condensate with Maxwell-like rheology (Extended Data Fig. 10b). At low driving frequencies, $\chi^*(\omega)$ approached the static spring constant, χ_0, which is a mark for surface tension. At higher frequencies, $\chi^*(\omega)$ augmented considerably, especially in its elastic part, $\chi'(\omega)$. The complex shear modulus of the droplets, $G^*(\omega)$, was finally obtained as follows[5]:

$$G^*(\omega) = \frac{\chi^*(\omega) - (1.25 + 4.36\theta_0^2)\gamma}{R(5.47\theta_0^5 - 29.28\theta_0^4 + 23.29\theta_0^3 - 5.08\theta_0^2 + 3.79\theta_0 - 0.02)} \tag{5}$$

Experimental data of the G^* modulus was fitted a non-fractional Maxwell element[2], from which parameters E (Pa) and eta (Pa·s), and the subsequent crossover frequency, $\omega_c = 1/(2\pi)E/\eta$ (Hz), was obtained. The crossover frequency defines the viscous- and elastic-dominant regimes characteristic of Maxwell materials. For $\omega < \omega_c$ the material flows, whereas for $\omega > \omega_c$ the material responds as a solid. A shift of ω_c towards lower frequencies indicates that the material responds with a more elastic behavior for a given frequency and has undergone maturation.

With the help of this formalism, we discovered that all condensates behaved as Maxwell materials with a strongly frequency dependent response (Extended Data Fig. 10b,c). Whereas pure MEC-2 condensates and condensates composed of mutant MEC-2 (R385H) and UNC-89 did not show a significant change in their mechanical parameters such as crossover frequency and plateau modulus, the condensates composed of wildype MEC-2 and UNC-89 showed a strong maturation. We found that the crossover frequency shifted to 3 times lower values after 24 h maturation from ≈3Hz to 1Hz, whereas the plateau modulus E slightly increased, even though only marginally in all compositions tested. This subtle increase in E may suggest that MEC-2 is distinct from the glass-like behavior measured previously[1]. In support for this observation is the fiber formation, which suggest the appearance of some degree of order during the spontaneous extrusion, which is incompatible with the assumption of a frustrated glass-like state. Indeed, the observation of the stiffening response could be due to increased crosslinks, especially

in the binary mixture composed of MEC-2 and UNC-89. These fibrillar structures exhibited a strong increase in stiffness and stress storing capacity, with almost no disspation during the indentation test. The force-time signal upon square actuation perpendicular to it by means of an optically-trapped microbead. For technical reasons, a rigorous microrheological analysis was impossible, but an ultrastructural analysis of the long range order in these fibers shall provide conclusive answers whether or not MEC-2 UNC-89 mixtures represents a glass or gel-like state.

How does a change in cross-over frequency affect cell signaling and mechanics? It offers a potentially unifying framework to understand how the ion channel kinetics are governed by the mechanical response of the viscoelastic environment. We suspect that for stimulation frequencies slower than the relaxation time constant, forces are effectively dissipated, as the material relaxes faster than the stimulation is propagated. If the material is part of the force transmission pathway, e.g. links the ion channel to the ECM or the cytoskeleton, these slow stimulations are dampened, which leads to an interruption of the mechanical transmission. Such mechanical frequency filters have been proposed to account for the frequency-selection in *C. elegans* touch[6, 7, 8]. Our values of the cross over frequency ($\approx$1Hz) in the mature binary droplets represents the lowest bound at which the elastic and viscous properties are equal. From this, we reason that effective force transmission shall only happen for faster stimulation, which is indeed seen in *C. elegans* touch. Our observation that the crossover frequency shifts to lower values in presence of UNC-89 suggest that the mature condensates behave as a solid, and thus conservative, over a larger frequency range. Thus, it offers a unifying principle by which the material environment of mechanical strain sensors become sensitive to external mechanical stimulations in a frequency dependent manner[6, 9, 10, 11]. It also may suggest a principle by which a regulated change in the viscoelastic properties enables a transient switch between rapidly adapting or slowly adapting mechanosensors. In this view, coacervation of different binding partners in the condensed phase may trigger viscoelastic changes with consequences on the force transmission and ion channel open/closing.

How does the maturation time course compare to the maturation of other condensates? We found that pure MEC-2 showed significant maturation after 48h which is significantly slower than the maturation of PGL-3 droplet measured with active microrheology in ref. [1] and much slower than the aging of SPD-

5[12]. We also found that the increase in relaxation time was accelerated in presence of UNC-89, which suggests that binding of the SH3 domain to the MEC-2 proline motif causes structural rearrangements between protein molecules within the condensate. Because our measurements on MEC-2 reveal higher viscosity and elastic modulus at high salt concentrations, the values of the surface tension in the order of 20-60 μN/m from our measurements is in agreement with values derived from FUS droplets[13]. We should note that the viscoelastic time scale measured in the rheology experiment characterizes the relaxation to an external, mechanical perturbation and hence does not necessarily coincide with the diffusion time scale in the FRAP experiment which is derived at equilibrium. As the importance of the rigidity of a material comes only to play under external load (e.g. touch), the optical tweezer and tension sensor experiments provide a more reliable readout for the rigidity transition. The diffusion coefficient thus provides information about the distinct mobility of the labeled MEC-2 in the naive and mature condensates, but cannot resolve differences of the viscoelastic relaxation of unstressed condensates once they are matured. Indeed, the fluctuation-dissipation theorem that relates thermal motion of a particle with the bulk mechanical properties does not necessarily hold in out-of-equilibrium materials such as glassy systems[14] and living cells [15, 16]. Thus, we shall emphasize here that an aging system is not at thermodynamic equilibrium, thus the relaxation time scale from the active microrheology and the FRAP recovery dynamics may not necessarily coincide[1].

2 Supplementary Note 2: Plasmid design and genome engineering

2.1 Expression of wild type and R385H *mec-2* and *unc-89* for *in vitro* purification

Wild type complementary DNA ranging from position 371 to 481 of MEC-2, which includes the C-terminal domain, was subcloned into a pCoofy expression vector (donated by Carlo Carolis lab) containing an N-terminal polyhistidine affinity tag and a NusA solubility tag for posterior MEC-2 purification, giving to pNS66. This plasmid was used as a template to incorporate the R385H single point mutation (*u26* allele) in MEC-2 by site-directed mutagenesis, giving to pNS73. The plasmid pNS66 was used as a template to mutate the prolines of the PRM motif into alanines (^{377}PPSLP^{381}$\rightarrow$^{377}AASLA^{381}; MEC-2ΔPRM) by site-directed mutagenesis, giving to pNS80. This plasmid was used as a template to

incorporate the R385H single point mutation (*u26* allele) in MEC-2 by site-directed mutagenesis, giving to pNS81.

Wild type complementary DNA encoding 61-128 amino acids of UNC-89, which includes the SH3 domain, was subcloned into a pET-24 expression vector (ordered from Twist Bioscience) with an N-terminal polyhistidine affinity tag for posterior UNC-89 purification, giving to pNS75. A previous version including 1-454 amino acids of UNC-89 led to unfolded purified protein.

2.2 Expression of wild type and R385H *mec-2*::mCherry *in vivo*

Wild type complementary DNA encoding full-length MEC-2 (A isoform) was subcloned into pBCN27[17] to replace puromycin resistance gene, and fused to mCherry, generating pMK8. Site-directed mutagenesis was used to introduce the R385H single point mutation (*u26* allele) in MEC-2, giving pMK9. A truncated version of MEC-2 (1-370 aa) was generated to delete the C-term from the pMK8 template giving pNS79. The three plasmids were integrated by the MosSCI method[18] in the EG6699 strain, that contains compatible MosSCI landing sites in Chr. II, leading to MSB87, MSB88 and MSB1151, respectively. MSB1151 was crossed to MSB99 to incorporate the *mec-2(u37)* allele (W119Stop) in the endogenous locus (Chr. X) leading to MSB1157. Overexpression of a region of the C-terminus (365-396 amino acids) of MEC-2 containing PRM was done by cloning MEC-2 wild type or R385H mutant from a gBlock under TRN-specific *mec-17*p, thus generating pNS10 and pNS12, respectively, and injecting them as extrachromosomal array.

2.3 Generation of the *u37* allele in *mec-2*

The *mec-2(u37)* allele (W119Stop), which introduces a premature stop codon, was reproduced by CRISPR/Cas9 genome editing as described in[19, 20]. Two crRNAs were designed to cut few base pairs before the target site together with a donor consisting of a ssODN with 35 basepair (bp) homology arms flanking the polyspacer adjacent motif (PAM) sequence, the desired single point mutation and 5 other silent mutations to facilitate the posterior screening of the edit by PCR. Briefly, the Cas9-crRNA-tracrRNA RNP complex and the homology repair template (HDR) were assembled in Mili-Q water, together with the Cas9 complexes and HDR for the marker gene *dpy-10*, to introduce the semi-dominant

cn64 allele. 20-30 young adult hermaphrodites were injected with the CRISPR mix and recovered onto individual plates. After 3 days cultured at 25ºC, the progeny was screened based on the dpy or roller phenotype and singled onto individual plates. Mothers were lysed, screened by PCR for the corresponding edit and verified by sequencing. Sequences of crRNAs and ssODN donors are provided in Supplementary Data Table 6.

2.4 Expression of *mec-2* in hypodermis

For *mec-2* expression in hypodermis, the *wrt-2* hypodermal promotor was amplified from genomic DNA (1376 bp) and *mec-2*::mCherry::coLOVpep was amplified from pNS13, both were cloned into pNMSB35 backbone giving pNS70. It was injected at 30 ng/ul leading to MSB991. pNS70 was used as a template to incorporate the R385H single point mutation in MEC-2 by site-directed mutagenesis, giving to pNS78. It was injected at 30 ng/ul leading to MSB1144.

2.5 Generation of the FRET constructs

The TSMod cassette containing mTFP, 40-amino acid-long flexible linker, mVenus and a TEV protease site, was amplified from pMG319[21] and inserted between 370-371 amino acids of MEC-2, leading to pNS2. This plasmid was used as a template to introduce the R385H mutation by site-directed mutagenesis to yield pNS24. The TSMod cassette was also inserted at the C-term of MEC-2 in the plasmid pMK13. These plasmids were integrated by the MosSCI method[18] in Chr II in strains with *mec-2(u37)* background in the endogenous copy (Chr X), generating MSB341, MSB357 and MSB74, respectively. The low FRET construct was made by replacing the TSMod cassette in pNS2 with a mTFP-TRAF-mVenus cassette derived from pMG352[21], which constitutively separates the donor and acceptor fluorophores, and it was injected as extrachromosomal array giving to MSB907. A TEV protease site was fused to mCherry through a spliced leader SL2 (gpd-2-gpd-3) under TRN-specific *mec-17* promotor, giving to pMK97. It was injected into MSB341 leading to MSB403. Expression of the FRET construct in hypodermal cells was made by replacing the promotor of pNS2 by *wrt-2*p from pNS70, giving to pNS82, which was injected to N2, generating MSB1173.

2.6 Promotor trapping of *unc-89*

The *unc-89* promotor expression vector, was generated by amplifying 4 kb upstream to *unc-89* gene and the first three exons, including the SH3 domain, from *C. elegans* genomic DNA (Supplementary Data Table 7). It was transcriptionally fused to GFP through a spliced leader SL2 (gpd-2/gpd-3), generating pNS49, which was injected in MSB87 animals, leading to MSB656.

2.7 Tagging of *unc-89* SH3 domain

The plasmid to tag UNC-89 SH3 domain (63-127 amino acids) was generated by amplifiying the SH3 motif from N2 genomic DNA and cloning it under the TRN-specific *mec-18* promotor from pMK105. It was fused to GFP with a 5 amino acids linker from IR83 giving to pNS41. For *spc-1* SH3 tagging, the *mec-17* promotor and *spc-1* SH3 motif were taken from the pMK32 backbone and fused to GFP with a 3 amino acids linker from pDD282 giving to pMK101. They were injected at 20 ng/ul giving to MSB493 and MSB544, respectively.

2.8 Insertion of TEV site in *mec-2*

Two TEV cleavage sites preceded by a flexilinker were inserted between amino acids 370-371 of MEC-2 by CRISPR/Cas9 genome editing. Two crRNAs were used to cut exon 9 of *mec-2* A isoform and a 134 bp ssODN was used to repair it and incorporate flexilinker::x2TEV, see Supplementary Data Table 6. It was done on N2, generating MSB1127. Animals were verified by sequencing. The resulting animals were touch sensitive (Fig. 1h) and were injected with TEV protease (pMK97) leading to MSB1141.

2.9 Mutation of PRM in *mec-2*

The mutation of the prolines in the PRM motif of MEC-2 to alanines (^{377}PPxxP^{381}$\rightarrow$^{377}AAxxA^{381}; MEC-2ΔPRM) in the endogenous locus of *C. elegans* was done by CRISPR/Cas9 genome editing, as descrived before, giving to MSB1236. Sequences of crRNA and ssODN are provided in Supplementary Data Table 6.

2.10 Generation of (mEGFP(loxP)::AID knock-in)*unc-89*

The Nested CRISPR/Cas9 genome editing[22] was used to knock-in mEGFP at the *unc-89* gene. Two crRNAs were used to cut the N-term of *unc-89* and it was repaired by 200 bp ssODN containing parts 1 and 3 of mEGFP including a loxP within a synthetic intron of the mEGFP, along with a flexible linker and a degron site (AID). A new PAM site and a protospacer sequence was inserted in the first fragment to allow the in-frame insertion of the remaining sequence mEGFP2, designed as an IDT gBlock. For the second step, the same universal crRNA mentioned in[22] was used to make the double stranded break (see Supplementary Data Table 6). The correct in-frame insertion of the full length mEGFP was sequence verified and correct UNC-89 expression was checked by green fluorescence expression in muscles. The knock-in was done on top of MSB87, generating MSB523.

2.11 Generation of split wrmScarlet *unc-89*

When we tagged the endogenous *unc-89* locus at the N-terminus with GFP (Extended Data Fig. 5c), we visualized that its expression was largely restricted to the muscles (Extended Data Fig. 5k). With the previous functional results in mind (Fig. 3a-c and Extended Data Fig. 5e,g), we hypothesized that UNC-89 is expressed in TRNs (Supplementary Data Table 1 and Extended Data Fig. 5b) in quantities that cannot be detected above background level if it is only tagged with a single fluorophore at the endogenous locus. Thus, we sought to visualize UNC-89 distribution using multiplexed split fluorescent protein complementation[23] by tagging the endogenous locus with 5x wrmScarlet(11) and co-expressing wrmScarlet(1-10) selectively in TRNs. To visualize an enhanced endogenous level expression of *unc-89*, it was used the multiplexed split fluorescence protein complementation[23]. 5 motifs of wrmScarlet barrel 11 separated by flexilinkers were inserted at the N-term of *unc-89* by CRISPR/Cas9 genome editing, see Supplementary Data Table 6. The resulting strain was injected with *mec-4*p::wrmScarlet 1-10 (pNS50) as extrachromosomal array in order to restrict the expression selectively in TRNs and led to MSB791.

2.12 Generation of the conditional and constitutive *unc-89* knock-out

unc-89 knock-out was generated by CRISPR/Cas9 genome editing using two crRNAs to cut exon 3 of *unc-89* and a 108 bp ssODN that led to a frame-shift and absence of the largest isoforms (a,b,e,f,k,l,m,n,o), see Supplementary Data Table 6. It was done on MSB523 mEGFP(loxP)::degron::*unc-89*, generating MSB590. Animals were verified by sequencing and by absence of green fluorescence in muscles. The resulting animals move normally but show a slight delay in development and body size (Extended Data Fig. 5d). These animals were crossed to MSB341 in order to assess FRET in a *unc-89* mutant background.

2.13 CRE/loxP and AID degradation

Tissue specific *unc-89* knock-out was generated by inserting an in-frame loxP site at the C-term of the *unc-89* largest isoforms by CRISPR/Cas9 genome editing. Two crRNAs were used to cut the C-term of *unc-89* and it was repaired by a 129 bp ssODN that carried a loxP and 5 silent mutations for posterior screening by PCR (see Supplementary Data Table 6). It was injected in MSB523 (mEGFP(loxP)::AID::*unc-89*) giving to MSB930. The correct in-frame insertion was sequence verified and correct *unc-89* expression was checked by green fluorescence expression in muscles. MSB930 was crossed to MSB926, which carries the TRN-specific *mec-17*p::CRE and the *mec-4*p::TIR[24] leading to MSB953. Alternatively, MSB930 was crossed to MSB933 which carries the panneuronal (*rgef-1*)p::CRE leading to MSB941.

3 Supplementary Videos

Supplementary Video 1. Organization and dynamics of MEC-2 in TRNs. Representative videos of the MEC-2::mCherry dynamic and static pools in touch receptor neurons: 4 ALM and 4 PLM neurons.

Supplementary Video 2. Dynamics of MEC-2 condensates *in vivo*. A representative video of a MEC-2 condensate undergoing deformation events during translocation along the neurite. Frame rate = 20 fps. Scalebar=2μm.

Supplementary Video 3. Dynamics of MEC-2 condensates *in vivo*. A representative video of a MEC-2 condensate undergoing fission events during translocation along the neurite. Frame rate = 20 fps. Scalebar=2μm.

Supplementary Video 4. Dynamics of MEC-2 condensates *in vivo*. A representative video of a MEC-2 condensate undergoing fusion events during translocation along the neurite. Frame rate = 20 fps. Scalebar=2μm.

Supplementary Video 5. MEC-2 dynamics using FRAP. Representative FRAP dynamics of the MEC-2::TEV::MEC-2 static pool and the C-terminally truncated construct after TEV co-expression.

Supplementary Video 6. Fusion dynamics of MEC-2 condensates *in vitro* Representative videos of the MEC-2 droplets undergoing fusion events *in vitro*.

Supplementary Video 7. Touch-induced calcium transients. Representative videos of a wild type and a MEC-2::R385H mutant animal expressing a GCaMP6s calcium reporter in TRNs. A two second buzz was delivered after 10s. Top images are the calcium sensitive, lower images are the calcium insensitive channel (tagRFPt).

Supplementary Video 8. Dual optical trap rheology. Representative video of the dual trap assay to measure the complex shear modulus. Left bead is firstly applying sinusoidal oscillations at increasing

frequencies $f = 0.5, 1, ..., 64$ Hz, and secondly applying a step indentations, while the right bead is static.

4 Supplementary Data Tables

Supplementary Data Table 1 List of candidate genes used for the RNAi experiment according to a preselection of *C. elegans* proteins with an SH3 domain[25] and proteins expressed in TRNs[26]. Included the library chosen for each clone and the result of the touch assays after RNAi knock-down (Mean±SD). N=40 animals; 5 touches each. *p*-values derived from non-parametric multiple comparison post-hoc Dunn's test with Bonferroni correction.

Supplementary Data Table 2 Fit parameters extracted from the rheological spectrum in Fig. 5. *p*-values derived from a pairwise comparison using a two-tailed t-test. Pure MEC-2 viscosity: 0 vs 24h; p_η=0.01; elastic plateau modulus: p_E=1.4e-4; crossover frequency: p_{ω_c}=0.2. Co-condensate MEC-2:UNC-89 viscosity: 0 vs 24h; p_η=1.8e-4; elastic plateau modulus: p_E=2.1e-4; crossover frequency: p_{ω_c}=4.7e-4. Co-condensate MEC-2 R385H:UNC-89 viscosity: 0 vs 24h; p_η=0.23; elastic plateau modulus: p_E=0.01; crossover frequency: p_{ω_c}=0.9.

Supplementary Data Table 3 Statistics for RNAi experiment (Fig. 3a) derived from non-parametric Kruskal Wallis test followed by a two-sided pairwise Dunn's test for multiple comparisons.

Supplementary Data Table 4 List of strains used in this study.

Supplementary Data Table 5 List of plasmids and sequences used in this study.

Supplementary Data Table 6 List of CRISPR reagents: crRNAs and ssODNs used in this study.

Supplementary Data Table 7 Genomic sequence of promoter and first three exons of *unc-89*. See Supplementary Note 2 and Extended Data Fig. 5b.

Supplementary Data Table 8 Table showing the predicted proline rich motifs[27] of the stomatin homologs indicated in Extended Data Fig 1 a-g. The italicized motifs represent a highly conserved proline, implicated in membrane association and topology[28]. While buried in the stomatin domain, these are

likely not accessible, and have therefore been filtered out. Note, MEC-2 has another highly conserved PRM, predicted to bind to canonical class 2 and non-canonical class 1 SH3 domains, highlighted in boldface.

References

[1] Jawerth, L., et al., Protein condensates as aging Maxwell fluids. *Science* **370**, 1317–1323 (2020).

[2] Bonfanti, A., Kaplan, J. L., Charras, G., Kabla, A., Fractional viscoelastic models for power-law materials. *Soft Matter* **16**, 6002–6020 (2020).

[3] Alberti, S., et al., A User's Guide for Phase Separation Assays with Purified Proteins. *Journal of Molecular Biology* **430**, 4806–4820 (2018).

[4] Català-Castro, F., Venturini, V., Ortiz-Vásquez, S., Ruprecht, V., Krieg, M., Direct force measurements of subcellular mechanics in confinement using optical tweezers. *Journal of Visualized Experiments* **2021**, 1–35 (2021).

[5] Jawerth, L. M., et al., Erratum: Salt-dependent rheology and surface tension of protein condensates using optical traps. *Physical Review Letters* **125**, 229901 (2020).

[6] Eastwood, A. L., et al., Tissue mechanics govern the rapidly adapting and symmetrical response to touch. *Proceedings of the National Academy of Sciences* **113**, E2471–E2471 (2015).

[7] Sanzeni, A., et al., Somatosensory neurons integrate the geometry of skin deformation and mechanotransduction channels to shape touch sensing. *eLife* **8**, 1–44 (2019).

[8] Katta, S., Krieg, M., Goodman, M. B., Feeling Force: Physical and Physiological Principles Enabling Sensory Mechanotransduction. *Annual Review of Cell and Developmental Biology* **31**, 347–371 (2015).

[9] Loewenstein, W. R., Mendelson, M., Components of receptor adaptation in a Pacinian corpuscle. *The Journal of Physiology* **177**, 377–397 (1965).

[10] Handler, A., Ginty, D. D., The mechanosensory neurons of touch and their mechanisms of activation. *Nature Reviews Neuroscience* **22**, 521–537 (2021).

[11] Bolanowski, S. J. J., Zwislocki, J. J., Intensity and frequency characteristics of pacinian corpuscles. II. Receptor potentials. *Journal of neurophysiology* **51**, 812–830 (1984).

[12] Woodruff, J. B., et al., The Centrosome Is a Selective Condensate that Nucleates Microtubules by Concentrating Tubulin. *Cell* **169**, 1066–1077.e10 (2017).

[13] Ijavi, M., et al., Surface tensiometry of phase separated protein and polymer droplets by the sessile drop method. *Soft Matter* **17**, 1655–1662 (2021).

[14] Crisanti, A., Ritort, F., Violation of the fluctuation-dissipation theorem in glassy systems: Basic notions and the numerical evidence. *Journal of Physics A: Mathematical and General* **36** (2003).

[15] Wilhelm, C., Out-of-equilibrium microrheology inside living cells. *Physical Review Letters* **101**, 1–4 (2008).

[16] Turlier, H., et al., Equilibrium physics breakdown reveals the active nature of red blood cell flickering. *Nature Physics* **12**, 1–8 (2016).

[17] Semple, J. I., Garcia-Verdugo, R., Lehner, B., Rapid selection of transgenic C. elegans using antibiotic resistance. *Nature Methods* **7**, 725–727 (2010).

[18] Frøkjær-Jensen, C., et al., Single copy insertion of transgenes in C. elegans. *Nat. Genet.* **40**, 1375–1383 (2008).

[19] Paix, A., Folkmann, A., Seydoux, G., Precision genome editing using CRISPR-Cas9 and linear repair templates in C. elegans. *Methods* **121-122**, 86–93 (2017).

[20] Arribere, J. a., et al., Efficient Marker-Free Recovery of Custom Genetic Modifications with CRISPR/Cas9 in Caenorhabditis elegans. *Genetics* **198**, 837–846 (2014).

[21] Krieg, M., Dunn, A. R., Goodman, M. B., Mechanical control of the sense of touch by β-spectrin. *Nature Cell Biology* **16**, 224–233 (2014).

[22] Vicencio, J., Martínez-Fernández, C., Serrat, X., Cerón, J., Efficient generation of endogenous fluorescent reporters by nested CRISPR in caenorhabditis elegans. *Genetics* **211**, 1143–1154 (2019).

[23] Goudeau, J., et al., Split-wrmScarlet and split-sfGFP: tools for faster, easier fluorescent labeling of endogenous proteins in Caenorhabditis elegans. *Genetics* **217** (2021).

[24] Das, R., et al., An asymmetric mechanical code ciphers curvature-dependent proprioceptor activity. *Science Advances* **7**, 1–20 (2021).

[25] Xin, X., et al., SH3 interactome conserves general function over specific form. *Molecular Systems Biology* **9**, 1–17 (2013).

[26] Lockhead, D., et al., The tubulin repertoire of C. elegans sensory neurons and its context-dependent role in process outgrowth. *Molecular biology of the cell* **27**, 3717–3728 (2016).

[27] Kumar, M., et al., The Eukaryotic Linear Motif resource: 2022 release. *Nucleic Acids Research* **50**, D497–D508 (2022).

[28] Kadurin, I., Hubert, S., Gründer, S., A single conserved proline residue determines the membrane topology of stomatin. *Biochemical Journal* **418**, 587–594 (2009).
