# Supplementary material for: A MEC-2/stomatin condensate liquid-to-solid phase transition controls neuronal mechanotransduction during touch sensing
Source: Nat Cell Biol. 2023 Oct 19;25(11):1590–9. doi: 10.1038/s41556-023-01247-0 (PMC10635833; doi:10.1038/s41556-023-01247-0)
Supplement: Supplementary file 2 — Reporting Summary [file 41556_2023_1247_MOESM2_ESM.pdf]

nature portfolio

Corresponding author(s): Michael Krieg

Last updated by author(s): 01/30/2023

Reporting Summary

Nature Portfolio wishes to improve the reproducibility of the work that we publish. This form provides structure for consistency and transparency in reporting. For further information on Nature Portfolio policies, see our Editorial Policies and the Editorial Policy Checklist.

Statistics

For all statistical analyses, confirm that the following items are present in the figure legend, table legend, main text, or Methods section.

n/a	Confirmed	
☐	☒	The exact sample size (*n*) for each experimental group/condition, given as a discrete number and unit of measurement
☐	☒	A statement on whether measurements were taken from distinct samples or whether the same sample was measured repeatedly
☐	☒	The statistical test(s) used AND whether they are one- or two-sided<br>*Only common tests should be described solely by name; describe more complex techniques in the Methods section.*
☒	☐	A description of all covariates tested
☐	☒	A description of any assumptions or corrections, such as tests of normality and adjustment for multiple comparisons
☐	☒	A full description of the statistical parameters including central tendency (e.g. means) or other basic estimates (e.g. regression coefficient) AND variation (e.g. standard deviation) or associated estimates of uncertainty (e.g. confidence intervals)
☐	☒	For null hypothesis testing, the test statistic (e.g. *F*, *t*, *r*) with confidence intervals, effect sizes, degrees of freedom and *P* value noted<br>*Give P values as exact values whenever suitable.*
☒	☐	For Bayesian analysis, information on the choice of priors and Markov chain Monte Carlo settings
☒	☐	For hierarchical and complex designs, identification of the appropriate level for tests and full reporting of outcomes
☐	☒	Estimates of effect sizes (e.g. Cohen's *d*, Pearson's *r*), indicating how they were calculated

Our web collection on statistics for biologists contains articles on many of the points above.

Software and code

Policy information about availability of computer code

Data collection	All data collection software (image acquisition software [HCImage version 4.4.2.7, µManager], NMR software (TopSpin version 4.0.8) , Optical tweezer LightAce software) is described in detail in the methods for each experiment and are open source or commercially available. No custom acquisition software was used.
Data analysis	Optical Tweezer and Calcium data were analyzed using custom-written Matlab scripts (Version 2021, Das et al, 2021); Ratiometric FRET data analysis was performed in IgorPro 6.37 as described in Krieg et al. 2014; and NMR data analyses were performed as described in detail using published open source software (CcpNmr version-3 and delta2D version-1). BLAST of protein sequences was performed using NCBI. FRAP analysis was performed with the easyFRAP online tool (Koulouras et al. 2018; version 1.11). Rheological analyses were perfomed using custom-written Matlab scripts (R2019b). Statistics and data plotting were done in R (version 4.2.2; 2022-10-31) with the package "ks" version 1.14.0 and Python (version 3). Image analysis was performed in ImageJ (version 1.53f51). All custom computer scripts are available on GitLab https://gitlab.icfo.net/rheo/Tweezers/droplet or supon request. No specialized standalone software was developed as part of this manuscript.

For manuscripts utilizing custom algorithms or software that are central to the research but not yet described in published literature, software must be made available to editors and reviewers. We strongly encourage code deposition in a community repository (e.g. GitHub). See the Nature Portfolio guidelines for submitting code & software for further information.

Data

Policy information about availability of data

All manuscripts must include a data availability statement. This statement should provide the following information, where applicable:

- Accession codes, unique identifiers, or web links for publicly available datasets
- A description of any restrictions on data availability
- For clinical datasets or third party data, please ensure that the statement adheres to our policy

The chemical shifts in the NMR data of MEC-2 and UNC-89 was deposited to Biological Magnetic Resonance Data Bank (https://bmrb.io/) under accession codes ID:51491 and ID:51490 respectively. Source data are provided with this study. The publicly available datasets used in this study are accessible through wormbase.org version WS289. Protein sequences were obtained from UniProt (O01761). Calcium imaging data of the animals under a mechanical stimulus has been deposited into zenodo.org under doi:10.5281/zenodo.8163972. All other data supporting the findings of this study are available from the corresponding author on reasonable request.

Human research participants

Policy information about studies involving human research participants and Sex and Gender in Research.

Reporting on sex and gender	n/a
Population characteristics	n/a
Recruitment	n/a
Ethics oversight	n/a

Note that full information on the approval of the study protocol must also be provided in the manuscript.

Field-specific reporting

Please select the one below that is the best fit for your research. If you are not sure, read the appropriate sections before making your selection.

☒ Life sciences ☐ Behavioural & social sciences ☐ Ecological, evolutionary & environmental sciences

For a reference copy of the document with all sections, see nature.com/documents/nr-reporting-summary-flat.pdf

Life sciences study design

All studies must disclose on these points even when the disclosure is negative.

Sample size	For behavioral data collection, a consistent number of 30 animals per genotype and condition was investigated. No a priori method or power analysis was applied to determine an optimal sample size. Sample size was chosen according to previous published standards (Krieg, NCB,2014), to remain experimentally feasible and avoid spurious overfitting.
Data exclusions	No statistical outliers have been identified.
Replication	Behavioral data is presented as a triplicate of 10 animals assayed in each experiment (each animal was tested 10 times). Replicates, in which control animals did not meet the a priori requirements (e.g. positive control not positive or negative control not negative such as a failure of a positive control group to respond to blue light in ATR condition in an optogenetics experiment) were not considered in the analysis. All attempts at replication were successful.
Randomization	Whenever possible, experimental conditions were randomized to avoid history effects in the optical tweezer mechanics experiments. To avoid adaptation of the animals to repetitive touch stimuli, a minimum interstimulus intervall of 10s was applied. Animals from different genotypes in each experiment were subjected to the same treatment conditions on the same batch of NGM agar plates to control for variations coming from the food source or subtle differences in the environment conditions. No specific order was followed in any of the experiments unless specifically indicated in the methods. All other samples were not randomized.
Blinding	Behavioral experiments (manual scored touch tests) were performed blinded with respect to genotype and animal treatment. Other experiments were not performed blind to treatment or genotype, as they were performed with computer-assisted analysis.

Reporting for specific materials, systems and methods

We require information from authors about some types of materials, experimental systems and methods used in many studies. Here, indicate whether each material, system or method listed is relevant to your study. If you are not sure if a list item applies to your research, read the appropriate section before selecting a response.

Materials & experimental systems

n/a	Involved in the study
☒	☐ Antibodies
☒	☐ Eukaryotic cell lines
☒	☐ Palaeontology and archaeology
☐	☒ Animals and other organisms
☒	☐ Clinical data
☒	☐ Dual use research of concern

Methods

n/a	Involved in the study
☒	☐ ChIP-seq
☒	☐ Flow cytometry
☒	☐ MRI-based neuroimaging

Animals and other research organisms

Policy information about studies involving animals; ARRIVE guidelines recommended for reporting animal research, and Sex and Gender in Research

Laboratory animals	Caenorhabditis elegans, N2 and mutants listed in Supplementary Table 4; Experiments were restricted to young adult hermaphrodites, unless otherwise indicated.
Wild animals	No wild animals have been used in this study.
Reporting on sex	Unless otherwise indicated, all experiments have been conducted on young adult hermaphrodite animals.
Field-collected samples	No field samples were used in this study.
Ethics oversight	Nematodes are excempt from ethics approval.

Note that full information on the approval of the study protocol must also be provided in the manuscript.
